# Supplementary material for: Splice-Junction-Based Mapping of Alternative Isoforms in the Human Proteome
Source: Cell Rep. Author manuscript; Available in PMC 2020 Jan 15. (PMC6961840; doi:10.1016/j.celrep.2019.11.026)
Supplement: 2 [file NIHMS1546469-supplement-2.zip › DF1/PXD010154/Heart/Heart_63_TTN_VEAPPPKVPEVPK.pdf]

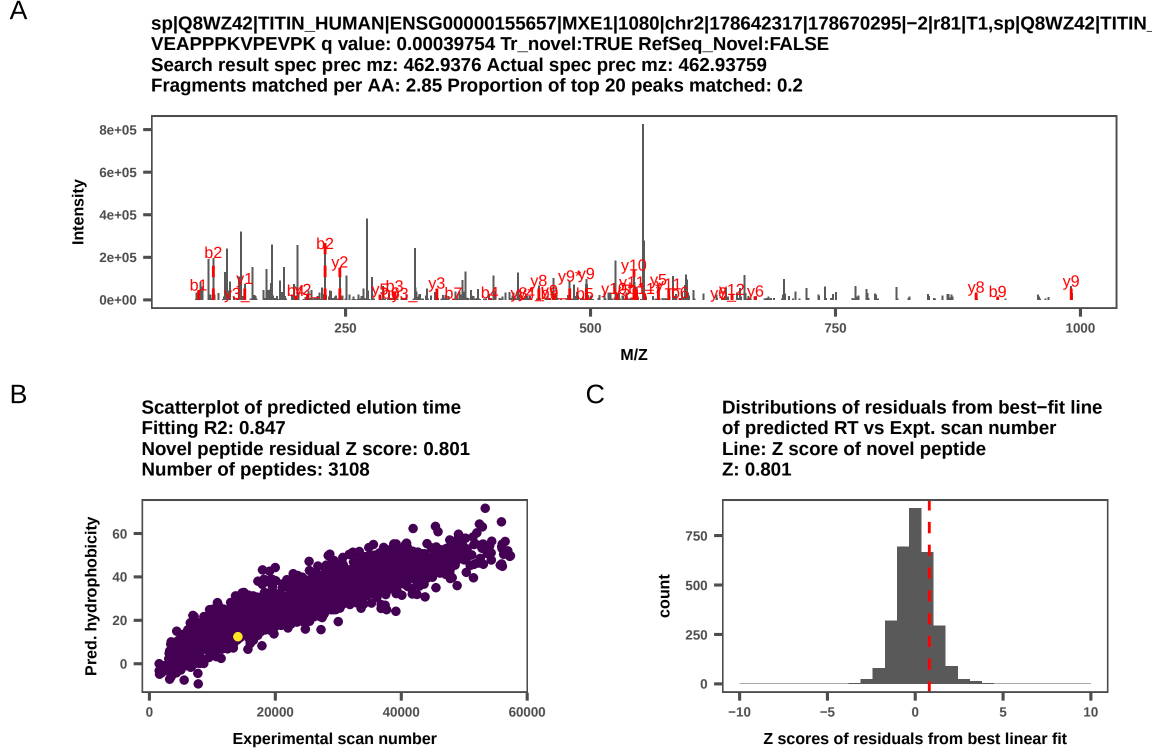
A
sp|Q8WZ42|TITIN_HUMAN|ENSG00000155657|MXE1|1080|chr2|178642317|178670295|−2|r81|T1,sp|Q8WZ42|TITIN_
VEAPPPKVPEVPK q value: 0.00039754 Tr_novel:TRUE RefSeq_Novel:FALSE
Search result spec prec mz: 462.9376 Actual spec prec mz: 462.93759
Fragments matched per AA: 2.85 Proportion of top 20 peaks matched: 0.2
Intensity
8e+05
6e+05
4e+05
2e+05
0e+00
250
500
750
1000
M/Z
B
Scatterplot of predicted elution time
Fitting R2: 0.847
Novel peptide residual Z score: 0.801
Number of peptides: 3108
Pred. hydrophobicity
60
40
20
0
0
20000
40000
60000
Experimental scan number
C
Distributions of residuals from best−fit line
of predicted RT vs Expt. scan number
Line: Z score of novel peptide
Z: 0.801
count
750
500
250
0
−10
−5
0
5
10
Z scores of residuals from best linear fit
